# Dissections of the Dead

**Published:** 1868-01

**Authors:** 


### ARTICLE VII.

### *Dissections of the Dead.*

By Prof. Gibbons.

As soon as people adopt rational views on this subject—as soon as they realize the fact that if physicians and students have no opportunity of dissecting the dead, the living must suffer—that themselves, their wives and their children must be the victims, then they will not only exact from the practitioner of Medicine and Surgery an adequate knowledge of Anatomy founded on practical dissections, but they will provide the means for dissection.

A few states of the Union, rising above the standard of Chinese legislation, have legalized dissections, by providing that the bodies of persons dying in public institutions without relatives or friends to claim them, shall be at the disposal of physicians for dissection, under proper conditions and restrictions. We have a law in California to that effect. Such statutes are salutary, for the reason that they remove all incentives to the desecration of the grave. With this status in force, the friends and kindred of persons interred in our cemeteries may banish from their minds all apprehension of disturbance of the bodies. Such apprehensions, however, are not well founded anywhere, under ordinary circumstances ; for the public institutions of large cities always furnish ample material for anatomical examination. Besides, the risk of detection is too great, and the consequences too serious, to admit of the invasion of private cemeteries.

It is a great mistake to suppose that practicing physicians and surgeons are in the habit of dissecting, and that "subjects" are in demand among them. The common people ought to know that dissections are not performed at the houses or offices of physicians ; that not one in fifty ever dissect a dead body after procuring his diploma ; that it is only in connection with the education of students in medical schools that cadavers are needed ; and that a very small number are sufficient for all purposes.

It is by no means creditable to the newspaper press, considered as an instrument for diffusing light and knowledge, that its influence is very generally directed so as to foster the foolish and barbarian prejudice against anatomical dissections. The story of a dead body in a box found on its supposed way to some doctor's garret, makes more noise than an earthquake. It is dressed up with every possible horror, and passed round through all the papers, as if to frighten people into the belief that a corpse is never safe in the grave, but that the doctors are prowling about all the cemeteries, like hyenas. There is no intention in this to impede scientific inquiry ; only, such items are too valuable to be thrown away in making up a newspaper.

Not many years ago a famous trial took place in Boston, the result of which appeared to depend in a great degree on the fact that the slayer, for the purpose of concealment, had cut up the body of the victim and burned a portion of it. Such a deed was enough to overshadow all palliative doubts in regard to the circumstances of the homicide, and shut out the prisoner from all hope of mercy.

The mutilation of the corpse was held up to the jury with telling effect.

The same irrational prejudice which would prohibit all dissections of the dead body, also interferes with examination after death, for the purpose of ascertaining the seat and nature of the disease. Physicians should strive to educate the popular mind on this point by making examination whenever practicable. There is but little difficulty in the intelligent classes of society. Reflecting persons have sense enough to know that diseases run in families, more or less, and that the inspection of the lungs, or the heart, or the stomach of one member of a family, dying with disease of those important organs, may furnish the means of saving the life of some of the surviving members. The fault is often with the physician himself, who makes no proper effort, or, worse still, no effort at all, to procure an examination, even in cases of great interest and importance. Sometimes the objections can be readily overcome by the clergyman of the family. Ministers of all denominations will almost invariably aid the physician in this respect.

If disease should invade the farm-yard and begin to carry off the cattle, how soon would the husbandman see that an examination be made of the dead cow or horse, for the benefit of the living! Even hogs and dogs would seldom be permitted to suffer for want of dissection of their dead comrades, to ascertain the cause of death. But when it comes to human beings, to the children and the family, a foolish prejudice interposes against their health and lives.

Persons are apt to entertain erroneous and often absurd notions as to the extent and manner of necropsic examinations. Care should be observed in asking consent to correct such errors. Sometimes it is not necessary to examine further than the abdomen or the chest. I have frequently overcome objections by urging the advantage to the preservation of the body, of removing the contents of the bowels, as it is here that decomposition commences. Where it is desirable to keep the body several days before interment, the introduction of an antiseptic into the cavities, which is a sort of embalming, may be absolutely necessary.

Let me urge the propriety of making post mortem examinations in all cases where consent can be obtained. To young physicians is this especially important. It familiarizes them with the use of the scalpel, and perfects their knowledge of Anatomy, to some extent. It imparts knowledge, positive or negative, in regard to disease. It familiarizes the popular mind to a great necessity of science.

Many important problems in Physiology, which could never have been solved in any other way, have been determined by experiments on the living bodies of inferior animals. Attempts have been made on the score of humanity, in England and America, to prevent vivisections. In the City of New York a society for the prevention of cruelty to animals has arraigned Prof. Dalton before the public on this ground, and also appealed to the Legislature to prohibit the practice. A French journal notices the movement, and remarks that the New York society says nothing about the many thousand cattle which are slaughtered every year in Brazil to supply the New York market with raw-hide whips.

Physicians have frequently sought to abate the prejudice against dissections by requesting that their own bodies should be dissected or examined after death. Old Dr. Monsey, who died in 1788, at the age of 95, the favorite physician of Chesterfield and Robert Walpole, went so far as to direct that his body be dissected, and the remains of his carcass, to use his own language, "be buried in a hole, or crammed into a box with holes, and thrown into the Thames." His body was actually dissected, according to his instructions, and a lecture given upon it to the students of Guy's Hospital. I have known many instances of physicians requesting post mortem examinations, for the purpose of determining satisfactorily the nature of their disease. One case of a very different character fell under my observation. A physician dying of an obscure organic affection, the necropsic investigation of which would have been highly instructive and useful, said to me with great emphasis, when he felt his end to be nigh—"Under no circumstances am I willing that my body shall be opened after death." His wish was complid with, of course; but the diploma of such a man ought to be cancelled.

Civilization demands that the lifeless body be treated with decent respect; but veneration for it belongs to Paganism. To burn it and preserve the ashes was once the highest honor. In modern times it is buried in the earth. The Chinese in California are content to secure the bones and transport them to their native land. These things are governed by usage. Our custom is to clothe the body carefully, and deck it with flowers, to lay it in a well-wrought casket; and then it passes forever from view. Its putrefaction and decay we do not then behold. If we did, the probability is that we should begin to inquire whether it would not be better to burn it and preserve the ashes in a sacred urn. I hope the day is not far distant when it will be considered a slight and a dishonor to a dead body to bury it, like a dog, without scientific inspection. Inert and lifeless, a tenement deserted, a form without the soul which so lately inspired it, it is nevertheless a miracle of Divine workmanship. And there is more respect shown to its Creator, in reading from it a lesson of the wisdom and skill of the Architect, than in committing it, unimproved, to silence and to dust.—*Pacific Med. and Surg. Journal.*

